# Critical Analysis of Preprints and Inquiry-Based Lessons Improve the Synthetic Biology Learning Experience

**DOI:** 10.1021/acssynbio.5c00014

**Published:** 2025-08-15

**Authors:** Guillermo Nevot, Marc Güell, Javier Santos-Moreno

**Affiliations:** † Department of Medicine and Life Sciences, 16770 Universitat Pompeu Fabra, 08003 Barcelona, Spain; ‡ ICREA, Institució Catalana de Recerca i Estudis Avançats, 08003 Barcelona, Spain

**Keywords:** Education, active learning, collaborative learning, synthetic biology, CRISPRi, preprints

## Abstract

Synthetic biology is a transformative field crucial to address global challenges. It is highly interdisciplinary, integrating different subjects beyond biology. Therefore, traditional lecture-based teaching methods often fall short in effectively covering the diverse and rapidly evolving advancements in synthetic biology. We developed active learning workflows for complementing classic theoretical lectures in universities to improve the synthetic biology learning experience. We used preprints as an educational resource for the students to critically analyze differences comparing manuscripts and the final published work. In addition, we designed a practical laboratory session where students had to infer the logic behind CRISPRi-based gene circuits that they assembled, thus engaging with every step of the design–build–test–learn cycle. Following these activities, 90% of the students reported having improved critical analysis skills and 80% felt that they had learned a wide range of synthetic biology concepts. These approaches demonstrate the potential of innovative teaching for synthetic biology, which helps students with both technical and soft skills at the same time and has the potential to be adapted to other fields.

## Graphical abstract

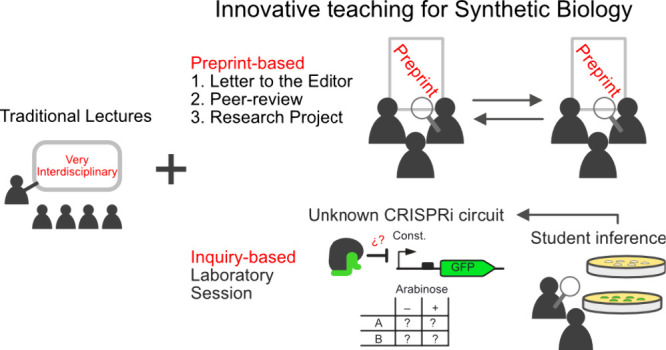

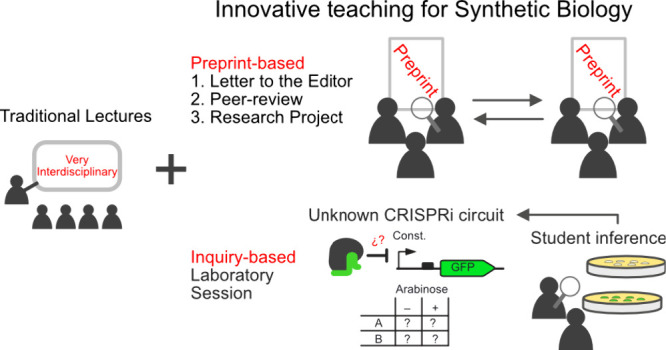

## Introduction

Globally, governments acknowledge that engineered biology will play a major role in future technological and economic advances,^1^ as demonstrated by the U.S. legislation on biomanufacturing^2^ and the European Commission communication for boosting biotechnology.^3^ Economic estimates report the impact of synthetic biology to span across industries like health and agriculture, with products already reaching consumers.^4^ These facts highlight the importance of synthetic biology to tackle global challenges. Thus, supporting students’ literacy and learning in this field becomes key for ensuring its long-term development and for promoting civic engagement and an informed social and ethical discussion regarding new synthetic biology technologies.

Synthetic biology is a field standing at the confluence of multiple scientific disciplines, including biology, biophysics, chemical engineering, computer science, control theory, and statistics, among others. While this diversity is one of the main strengths of synthetic biology, teachers and students are required to leverage knowledge from a wide range of disciplines, which poses a challenge when designing course content.^5^ Such a challenge is even greater in most life sciences university degrees, where synthetic biology is not a core component of the curriculum.

Traditionally, instructors have directly transferred their knowledge in a lecture-based, teacher-centered approach where students receive the information passively. In contrast, active, student-centered learning provides students with the opportunity to apply concepts as they learn. These approaches have been demonstrated to be more effective in promoting learning and show important advantages, including increased knowledge and the development of creativity, critical thinking, enhanced problem solving, and interpersonal and collaborative skills.^6–8^ Within synthetic biology, the iGEM competition is a clear example of the benefits of active learning for students.^9^ However, not all universities have the means to participate in such activities, which emphasizes the need to find alternative active learning approaches for synthetic biology within the curricula of university degrees. In this context, an active learning approach for a synthetic biology university course requires students to link concepts taught in class with their actual implementation in real examples. Students need to learn how to actively navigate concepts from different disciplines and apply them across scales.^10^ Making students evaluate synthetic biology research papers offers a learning opportunity to actively interconnect how concepts taught in class are actually implemented for real applications.

Preprints are manuscripts that have not undergone peer review. The open publication of preprints in dedicated servers has dramatically impacted the scientific publication process, as demonstrated by their growing popularity.^11^ In class, the opportunity of looking at “under construction” scientific works offers a unique learning experience for students. For instance, the critical assessment of preprints helps students gain insights into data presentation and improve academic writing skills.^12^

In this pilot study, we designed a tripartite group activity aimed at teaching synthetic biology to undergraduate biomedical engineering students. The learning objectives of this activity were (1) to interconnect synthetic biology concepts across scales and disciplines, (2) to foster critical and reflective thinking through in-depth analysis of real scientific articles, (3) to stimulate creative thinking toward application development within a specific synthetic biology discipline, (4) to promote cooperative work, and (5) to improve both written and oral communication skills. To achieve these goals, we first tasked students with writing a critical report of a synthetic biology preprint manuscript. In a second phase, students were asked to evaluate peers’ works and then to reflect on the critiques they received back from their peers. Finally, the third assignment consisted of designing a new research proposal and presenting it in a poster session. These three activities had an adequate workload and helped the students better grasp synthetic biology concepts, according to students’ feedback. To consolidate their learning and to help students correlate experimental results with the underlying molecular protocols, we combined standard lectures and this tripartite group activity with an inquiry-based lab session centered around CRISPR interference (CRISPRi)-based synthetic gene circuits.

## Methods

### Course and Student Description

The activities described here were purposed for the Advanced Synthetic Biology course, a three-month optional course worth 4 ECTS (European Credit Transfer System, a unified credit system to standardize academic workload across EU higher education) in the third or fourth year of the Biomedical Engineering degree at Universitat Pompeu Fabra. The course was given to 26 students, and the aim was to teach synthetic biology in depth for them to learn how to engineer living systems. The course contained classic theoretical lectures (43% of total hours) covering different aspects of synthetic biology, a tripartite activity (25%) comprising three assignments, and a guided lab session (32%).

### Preprint-Based Learning Activity Description

For this activity, students were gathered in five groups of five or six members each and offered a list of nine preprints spanning a wide range of landmark synthetic biology disciplines (Table S1); hence, we called this activity the “preprint-based activity”. The activity was then structured in three main assignments (Figure 1A). First, students were asked to write a Letter to the Editor (Assignment 1). Then the submitted letters were handed out to other groups, who were instructed to carry out a peer review and to write an Editorial Assessment, in which they judged the quality of the initial critique and its potential for publication (Assignment 2). Finally, groups were asked to come up with an innovative Research Project solving a key limitation of the original preprint or introducing a new application of the evaluated technology (Assignment 3). All these activities were made publicly available at https://advanced-synthetic-biology.super.site/ for students to showcase their work in front of classmates or external participants as well as in their CVs.

**Figure 1:**
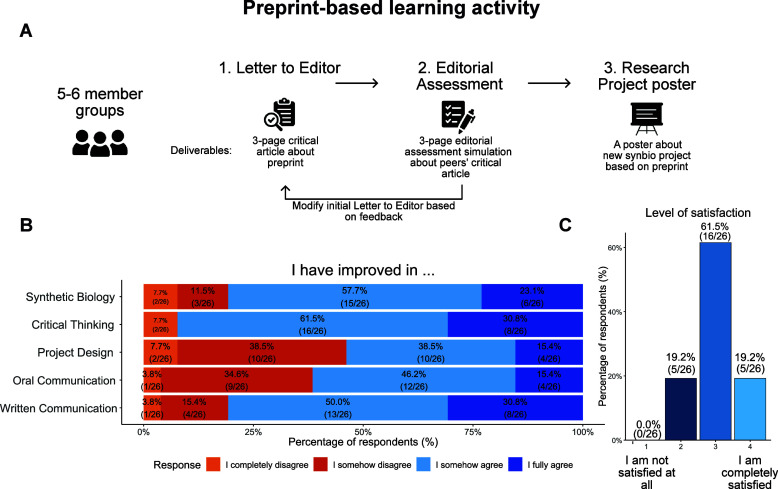
Critical analysis of preprints improves learning experiences. (A) Representation of the workflow for the tripartite preprint-based learning activity. (B) Responses of class participants to a survey asking whether they agree or not with having improved each designated ability. (C) Survey responses from class participants evaluating the level of satisfaction with the course on a 1 to 4 scale, where 1 stands for “Not satisfied at all” and 4 stands for “I am completely satisfied”.

The guidelines of the Letter to the Editor required the groups to criticize in a constructive manner the research scope, the data collection, and/or the result interpretation as well as related economic or societal aspects. For this assignment, students were given 16 days. The letter had to be written with a maximum length of 3 pages in a two-column format, with the possibility to include figures (Supporting Material S1).

The Editorial Assessment simulated the evaluation of the Letter to the Editor developed in the previous phase in order to make a decision regarding its rejection or acceptance. Students had to write peer-review reports and act as editors that summarized reviewers’ comments and proposed an editorial decision. They were given 11 days to perform this assignment in the form of a 2–3 page report with a specific format, which included a summary of the Letter to the Editor, two simulated reviews, and a reasoned editorial decision (Supporting Material S2).

Following the editorial assessment, students were tasked to reflect on the comments received and on their own Letter to the Editor and to come up with a new innovative Research Project to solve potential limitations that they may have identified, to ask new scientific questions, or to apply the technologies described in the preprints into new applications. These proposals were displayed in free-format posters during a poster session involving the whole class. Students could vote for their favorite poster, and instructors assessed all posters within the session. Students were given 27 days to prepare their posters.

Finally, student groups were provided with feedback from the instructors for the three assignments of the activity.

### Inquiry-Based Laboratory Session Description

Students worked in pairs within the laboratory for three sessions of 4 hours on consecutive days. The objective was to build CRISPRi-based gene circuits whose behavior could be evaluated thanks to a GFP reporter (Figure 2A,B). These CRISPRi circuits make use of a catalytically dead version of Cas9 (dCas9) that, in combination with a guide RNA (gRNA) directed against a promoter region, prevents the binding of the RNA polymerase, therefore blocking gene expression. Although all genetic parts were presented up front, the tubes were labeled with nondescriptive tags (e.g., “Y” or “Z” instead of “gRNA1” or “gRNA2”), and therefore, the students selected them in a blind manner without knowing which exact circuit they were attempting to build. The protocol was identical for all students independent of the gene circuit they were building, as we used a modular Gibson assembly scheme.^13^ Students followed a written script and instructors’ directions to carry out experiments aiming to (i) assemble the gene circuits using Gibson assembly, (ii) transform them into electrocompetent bacteria prepared by themselves, and (iii) measure the qualitative GFP signal of the transformants using a UV lamp. With the results in hand, they were able to infer the function of the parts that they had initially selected and of those selected by their classmates, which enabled them to unequivocally determine the identity of the synthetic gene circuits that they had been building. A complete description of laboratory methods, reagents, and instructions provided to the students can be found in Supporting Material S3. Although prior knowledge of molecular biology is recommended for students participating in this activity, the protocol can be adapted to different audiences (e.g., high school students) following a similar simplified theoretical explanation but keeping the same laboratory items. All plasmids used during the lab session are available through Addgene (#140664, 124421, and 238993–239002).

**Figure 2:**
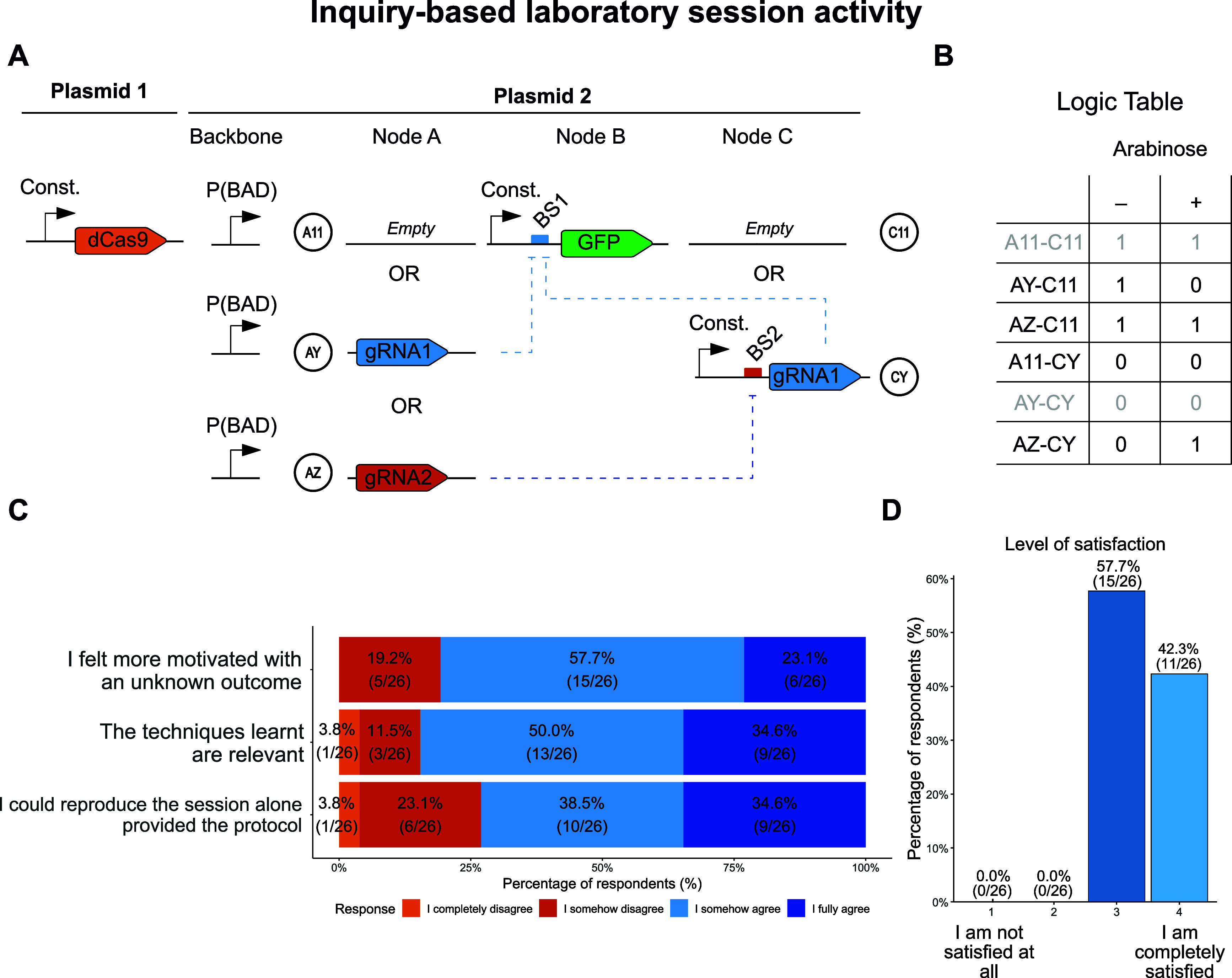
An inquiry-driven practical laboratory session improves perceived learning outcomes. (A) Schematics of the CRISPRi gene circuit components used in the session. Bacterial strains harbored a plasmid with constitutive dCas9, and students were tasked to build the second plasmid by combining three transcriptional units (nodes): node B was fixed, while several alternatives existed for nodes A and C, resulting in different circuits depending on the exact combination of nodes chosen. Students were aware of all these alternatives, but they ignored the exact identity of the parts they were working with, i.e., the node A used by any given group could be empty (A11) or contain gRNA1 or gRNA2 (AY or AZ, respectively); similarly, node C could be empty (C11) or contain a constitutive promoter upstream of a binding site for gRNA2 followed by gRNA1 (CY). (B) Truth table of the possible GFP fluorescence outcomes of all possible gene circuits. Combinations in gray were not allowed, to ensure an unambiguous outcome for the subsequent inference of the circuit identity. (C) Responses to a survey asking whether they agreed or not with the statements displayed. (D) Survey responses from class participants evaluating how satisfied they were with the course on a 1 to 4 scale, where 1 stands for “Not satisfied at all” and 4 stands for “I am completely satisfied”.

### Data Collection and Analysis

Students were asked to fill out an online questionnaire at the end of the poster session, i.e., once all group activities and lab sessions had been completed. This questionnaire consisted of five statements on a Likert scale^14^ and three questions to evaluate satisfaction and workload for the tripartite preprint-based learning activity and three statements on a Likert scale together with one satisfaction question to evaluate the lab sessions (Table S2). The answers required email registration to ensure that no duplicate answers were taken, but the collected data were anonymous and could not be traced back to the original respondents. Given the anonymous nature of the survey, no data protection provision was needed. All graphs and analysis were performed using R.

## Results

### Critical Analysis of Preprints Improves the Synthetic Biology Learning Experience

In previous years, the students reported that the theoretical content of the Advanced Synthetic Biology course was highly interdisciplinary, making it challenging for them to fully grasp the concepts behind different implementations and their corresponding limitations. For this reason, we designed a tripartite activity where groups of five students had to (1) write a critique of a synthetic biology preprint in the form of a Letter to the Editor, (2) peer review a critique from another group in the form of an Editorial Assessment, and (3) participate in a poster session presenting a new Research Project (Figure 1A).

For the Letter to the Editor elaboration, we preselected a list of preprints spanning many disciplines of synthetic biology in an effort to cover the wide range of technologies comprehended in this field. These included gene drive, CRISPR, cell-free transcription–translation systems, gene circuits, minimal cells, genetic code refactoring, biocontainment, and biomaterials (Table S1). All of the articles except one had a published version that the students could compare with the preprint version to find flaws or updates, serving as a starting point for their critique letters. We only explained what preprints are after the first Letter to the Editor had been submitted, thereby letting them get familiar with the differences on their own. As examples, we also provided articles published within the “Matters Arising” section of the journal *Nature*. “Matters Arising” are commentary articles on original research papers published within the past 18 months in some of the *Nature* journals. We asked the students to emulate these articles by crafting a similar commentary in the form of a Letter to the Editor.

From the selected preprints and the provided template (See Methods and Supporting Material S1), students elaborated a three-page Letter to the Editor and were able to articulate arguments focusing on a wide range of topics, from precise methodological claims to broad implications of the studies as well as challenges related to the economic viability or sustainability.

Then students received a Letter to the Editor from another group and had to elaborate a three-page editorial report with a provided template (See Methods and Supporting Material S2). In this report, students analyzed the letter from the point of view of reviewers and then summarized the different views from the editor’s perspective, recommending or not the publication of the Letter to the Editor. This step enabled students not only to learn about different synthetic biology disciplines but also to compare their arguments and reasoning with those of their colleagues. Students were allowed to modify their original work after receiving peers’ feedback and before the final evaluation by the instructors.

Following preprint analysis and subsequent peer evaluation, students were tasked to design an innovative Research Project. This assignment was instructed to build upon the technology described in the preprint that they had previously analyzed. The goal was either to develop an application of the technology or to tackle a new research question using that technology, and the desired format was that of a scientific poster. Poster sessions are an integral part of conferences and a valuable teaching resource. In our case, the poster session had two specific learning objectives: (1) to explain in a concise, semistructured, and interactive way a project in front of a network of (scientific) colleagues and (2) to engage in group discussions that include constructive criticism and questions to gather missing or desired information.

Overall, this part of the activity helped the students compare their ideas, share impressions on their arguments, receive oral feedback from their proposed projects, and promote in-depth scientific discussions. As the students had to personally select their favorite posters, this selection also encouraged them to evaluate the viability of the peers’ research proposals. Ten students (38%) actively highlighted the poster session as their preferred part of the tripartite activity when prompted with a free-format question.

After having completed the Letter to the Editor, the Editorial Assessment, and the Research Project poster presentation, 80% of the students reported that they increased their knowledge about synthetic biology (Figure 1B), and more than 90% of them also declared that they improved their critical analysis of research papers (Figure 1B). Additionally, this activity also contributed to students’ written skills, as reported by more than three-quarters of the class (Figure 1B).

One of our main concerns regarding this activity involved having too much workload imposed on the students due to the three assigment deliverables required. For this reason, we limited the number of pages and established big enough groups to ensure that tasks were distributed among the members. Additionally, at least 2 hours of class time for each assignment deliverable were dedicated to ask questions to and seek advice from the instructors. As a result, 62% of the class considered the workload to be low or just right, whereas 27% qualified it as high and only 11% reported the workload to be too high (Figure S1). In general, the satisfaction with this activity was high, with more than 80% of the class considering it positive (Figure 1C).

Finally, most students perceived that this activity helped them improve their oral presentation skills (Figure 1B). Although 80% of the students reported a better understanding of synthetic biology concepts (Figure 1B), only half of them felt confident to apply those concepts to the design of new synthetic biology projects (Figure 1B).

### Lab Sessions with Unknown Outcomes Improve Students’ Engagement and Learning

The Advanced Synthetic Biology course plan includes 12 hours of dedicated lab sessions. In spite of the limited time, we aimed at designing an inquiry-based lab session that includes aspects of all four phases of the design–build–test–learn (DBTL) cycle using CRISPRi-based gene circuits as a paradigm. Using a Gibson-based modular assembly approach,^13^ students cloned the CRISPRi circuits starting from their constituent parts (*build*) (Figure 2A), transformed them into homemade competent bacteria, and assessed their behavior in the absence or presence of the circuit inducer (*test*). Neither the identity of the constituent parts nor that of the final circuits was revealed to the students initially, forcing them to guess part and circuit identities during the lab session based on the observed circuit behavior. This was used as a surrogate of the *design* phase (Figure 2B)—indeed, the students had to design all possible circuits that could be constructed with the available parts, determine their theoretical behaviors, and compare them with the experimental behaviors to unambiguously identify all circuits and parts. Finally, the students *learned* about the circuits by comparing their results to the theoretical expectations: correct, incorrect, ambiguous, or missing results all provided learnings to the students that could potentially be incorporated in a prospective iteration of the DBTL cycle.

The great majority of the students (81%) felt more motivated by having an unknown outcome of the lab session (Figure 2C). At the same time, 73% of students reported being able to repeat the whole process alone if provided with the protocol (Figure 2C). Most of them also considered that the techniques they used are relevant for the field of synthetic biology (Figure 2C). These impressions were also reflected in the overall satisfaction, where all class students considered the activity positive (Figure 2D). Furthermore, 57% considered the lab sessions useful for their future career (Figure S2).

## Discussion

As teachers of the Advanced Synthetic Biology course, we had noticed that students struggled to incorporate synthetic biology concepts from pure theoretical lessons. These lessons usually cover a wide and multidisciplinary range of topics, making it difficult for the students to grasp the details involved in the implementation of each technology. While students could recall the concepts taught in class, they were not comfortable when asked to predict outcomes that required them to interconnect said concepts. In addition, the Biomedical Engineering degree in our university includes an interdisciplinary combination of mathematics and engineering subjects, where molecular and cell biology represent a smaller fraction of the curriculum. This academic plan demands that students embrace a diverse range of concepts, potentially challenging their ability to achieve a profound understanding in every specific discipline. To address this gap, we turned to preprints as a way, first, to encourage students to dive deep into a synthetic biology discipline while actively engaging with the material; second, to propose critical arguments and evaluate peers’ work; and third, to build upon the generated knowledge to create a new Research Proposal. We envisioned that this activity would help students navigate vertically across scales and horizontally across disciplines by taking a real synthetic biology implementation as a starting point. For example, in the study from Jung et al.,^15^ we expected students to reflect on the molecules and transcription factors selected for detection, the specific topology used for reporting, the actual deployment using cell-free systems, and the implications and importance of contaminant water detection. Indeed, students that selected this article included arguments demonstrating that they had looked at these four scales, from molecular to environmental levels, suggesting that they were able to zoom into one of the proposed disciplines and carefully examine it. Although initially students had to focus on one of the selected manuscripts (and thus on one synthetic biology discipline), this preprint-based learning activity also included a peer review assignment for the students to connect what they had learned to different disciplines described in other manuscripts.

Preprints offered a great learning opportunity. The availability of open peer review reports as well as the published version of the manuscripts served students as a starting point for their critical arguments, as they could see the changes made by the authors themselves before the final publication. At the beginning of the learning process, most of the students were not aware of the existence of preprint servers and of the differences between these manuscripts and the final published versions. We took advantage of this situation to establish a productive failure approach that helped us highlight the nature of preprints.^7^ Students elaborated their critiques without a clear explanation of what a preprint is, and we only explained the differences between preprint manuscripts and publications later (after the first submission of the Letter to the Editor), thus letting students initially explore preprints as part of their process of finding critical arguments. Although this preprint-based learning activity could be carried out with the final printed version of the articles, we believe that using manuscript preprints enriches the students’ view of the publication process and facilitates their critical analysis process. In addition, the design of the activity also encouraged them to value their own opinion and to raise relevant aspects related to the technologies, such as societal or economic implications. At the same time, students were held accountable for their work by having to submit their three assigment deliverables to the course website (https://advanced-synthetic-biology.super.site/). This resource was also useful to enable conversations between students, and it helped them compare how they differently approached the same assignments. The poster session was also key to building a community within the class and enhancing the critical spirit among peers. Students engaged very actively when presenting their posters and committed to scientific discussion.

Most of the students welcomed these activities and appreciated the effort to improve their learning experiences, actively contributing to shape it. Similarly, most of the students were clearly satisfied with the outcome of both activities and would recommend the course again (Figure S3). We should note that our analysis is based on instructors’ pedagogical experience and students’ perception, limiting our assessment of students’ learning and skill development. However, we observed that students increased their understanding of synthetic biology as a field, developed their critical and creative thinking, and improved their written and oral communication skills, therefore fulfilling our initial learning objectives.

Practical teaching of laboratory sessions has traditionally followed a recipe-like structure with defined outcomes. To encourage student commitment in these sessions, we explored an inquiry-based laboratory session where the outcome was unknown. This approach led the students to question every step of the process as well as the outcome obtained at each of them and to consider the technical implications of the methodology, in this case CRISPRi. Additionally, the use of a modular design also showed them how synthetic biology techniques focus on standards and processes that occur across multiple scales, allowing us to introduce these concepts in a practical manner. Similar approaches have also proven useful in the teaching of other life science disciplines.^16^ Here we argue that undefined outcomes improve student engagement also in the context of synthetic biology.

Engineering biology is becoming increasingly relevant as a way to address many global challenges. Thus, education in this field will be key to training future professionals and informing the civic society. We propose a methodology to balance the interdisciplinary nature and the deep technical understanding required for synthetic biology. For instance, designing gene circuits requires both creative problem solving and deep examination of the implementation—indeed, a few nucleotides in a poorly designed gRNA can lead to off-target binding. The proposed activities have been designed to help students navigate those challenges by complementing broad-topic theoretical classes with an active learning approach to delve into the specifics of several synthetic biology disciplines. We hope that this case example will be useful for other instructors willing to implement innovative approaches in the teaching of synthetic biology in order to improve both technical and soft skills among students. We provide all teaching materials in the , and plasmids for the laboratory sessions are available through Addgene (#140664, 124421, and 238993–239002).

## Supplementary Material









## Notes

A table with all preprints used in the preprint-based learning activity, with the title, reference of the manuscript, reference of the final published version, and the synthetic biology discipline covered; a table with the questions the students were asked to evaluate the activities; a table with the plasmids used for the laboratory sessions; a graph representing the students’ responses regarding how useful they perceive the lab sessions to be; a graph representing the students’ responses on how likely they would recommend the course to other students; a graph representing the students’ perception regarding the activity workload (PDF)
Letter to Editor template given to students for the first assignment of the preprint-based learning activity (PDF)
Editorial assessment template given to students for the second assignment of the preprint-based learning activity (PDF)
Teaching protocol for instructors wishing to reproduce the activity in their respective centers, in which all the laboratory session steps are explained in detail (PDF)
