# Supplementary material for: Critical Analysis of Preprints and Inquiry-Based Lessons Improve the Synthetic Biology Learning Experience
Source: ACS Synth Biol. 2025 Aug 15;14(8):2878–84. doi: 10.1021/acssynbio.5c00014 (PMC12362600; doi:10.1021/acssynbio.5c00014)
Supplement: Supplementary file 1 [file sb5c00014_si_001.pdf]

Supporting Information

Critical analysis of preprints and inquiry-based lessons improve synthetic biology learning experience

Guillermo Nevot[1*], Marc Güell[1,2] and Javier Santos-Moreno[1*]

[1]*Department of Medicine and Life Sciences, Universitat Pompeu Fabra, Barcelona, 08003 Spain*
[2]*ICREA, Institució Catalana de Recerca i Estudis Avançats, Barcelona, 08003 Spain*

*Correspondence to Guillermo Nevot (guillermo.nevot@upf.edu) and Javier Santos-Moreno (javier.santos@upf.edu)

This PDF file includes:

Supporting tables

Supporting Table S1.- Preprints and published articles used in this study.
Supporting Table S2.- Self-evaluation questionnaire for the participants.
Supporting Table S3.- Plasmids used for the laboratory sessions.

Supporting figures

Supporting Figure S1.- Students' responses about the usefulness of the laboratory sessions for their future career.
Supporting Figure S2.- Students' responses about course recommendations.
Supporting Figure S3.- Students' responses about the workload of the tripartite preprint-based activity.

Supporting Tables

Supporting Table S1.- Preprints and published articles used in this study.

Title	Reference preprint	Reference paper	Synthetic Biology discipline
Recording Gene Expression Order in DNA by CRISPR Addition of Retron Barcodes	(Bhattarai-Kline et al. 2022)	(Bhattarai-Kline et al. 2022)	DNA recording
CRISPR-Cas12a Target Binding Unleashes Indiscriminate Single-Stranded DNase Activity	(Chen et al. 2017)	(Chen et al. 2018)	CRISPR-based nucleic acid detection
Cell-Free Biosensors for Rapid Detection of Water Contaminants	(Alam et al. 2019)	(Jung et al. 2020)	Cell-free systems and biosensors
Design, Mutate, Screen: Multiplexed Creation and Arrayed Screening of Synchronized Genetic Clocks	(Lezia, Csicsery, and Hasty 2021)	(Lezia, Csicsery, and Hasty 2022)	High-throughput screening, logic circuits
Engineering Species-like Barriers to Sexual Reproduction	(Maselko et al. 2016)	(Maselko et al. 2017)	Gene drive
Evolution of a Minimal Cell	(Moger-Reischer et al. 2021)	(Moger-Reischer et al. 2023)	Synthetic minimal cell
A Swapped Genetic Code Prevents Viral Infections and Gene Transfer	(Nyerges et al. 2022)	(Nyerges et al. 2023)	Codon refactoring
Rational Design of Evolutionarily Stable Microbial Kill Switches	(Stirling et al. 2017)	(Stirling et al. 2017)	Biocontainment (kill switches)
Self-Pigmenting Textiles Grown from Cellulose-Producing Bacteria with Engineered Tyrosinase Expression	(Walker et al. 2023)	(Walker et al. 2024)	Engineered Living Materials

Supporting Table S2.- Self-evaluation questionnaire for the participants.

Number	Question	Subquestion	Answer Options
1	What is your level of satisfaction regarding the preprint-based learning activity?		I am not satisfied at all, 1, 2, 3, 4, I am completely satisfied
2	What is your level of satisfaction regarding the practical lab session?		I am not satisfied at all, 1, 2, 3, 4, I am completely satisfied
3	How likely is it that you recommend the course to other students?		Not likely at all, 1, 2, 3, 4, Very likely
4	How much do you agree with the following statements regarding the preprint-based learning activity?	I have improved my critical assessment of research articles	I completely disagree, I somehow disagree, I somehow agree, I fully agree
		I now know more about synthetic biology	I completely disagree, I somehow disagree, I somehow agree, I fully agree
		I have improved my oral communication skills	I completely disagree, I somehow disagree, I somehow agree, I fully agree
		I have improved my written communication	I completely disagree, I somehow disagree, I somehow agree, I fully agree

Number	Question	Subquestion	Answer Options
		I feel confident to design a successful synthetic biology project	I completely disagree, I somehow disagree, I somehow agree, I fully agree
5	Rate the following aspects of the preprint -based learning activity?	Activity workload	Too low, Low, Just right, High, Too high
6	How much do you agree with the following statements regarding the practical lab sessions?	The fact that I had to infer the outcome helped me to keep motivated	I fully agree, I somehow agree, I somehow disagree, I completely disagree
		I feel that the techniques learnt are relevant for the field of synthetic biology	I fully agree, I somehow agree, I somehow disagree, I completely disagree
		I fully understood all the concepts and I could easily repeat the procedures provided I am given the protocols	I fully agree, I somehow agree, I somehow disagree, I completely disagree
7	How useful do you think these practical lab sessions will be for your future career?		Not useful at all, 1, 2, 3, 4, Very useful
8	What are two things that you would like to keep in the course? Why?		Open-ended
9	What are two things that you would like to remove or improve in the course? Why?		Open-ended

Supporting Table S3. - Plasmids used for the laboratory sessions.

Name	Addgene ID	Resistance	Ori	Intended use	Comments	Source
pJ1996_v2	140664	Spectinomycin	CloDF13	Required for pEND circuits to operate as designed	Contains dCas9 and Csy4	Santos -Moreno et al., Nat. Commun. 2020, 11, 2746
pC-0	124421	Kanamycin	ColA	Empty backbone where Nodes A, B and C are inserted	Contains AraC, as well as P(BAD) upstream of the MCS	Santos -Moreno & Schaerli, ACS Synth. Biol. 2019, 8, 7, 1691–1697
pNA-011	238993	Spectinomycin	pUC	Empty Node A that can be used for the assembly of pEND-11Y	Empty	This study*
pNA-Y	238994	Spectinomycin	pUC	Node A carrying sgRNA-1 that can be used for the assembly of pEND-Y11	Contains sgRNA-1	This study*
pNA-Z	238995	Spectinomycin	pUC	Node A carrying sgRNA-2 that can be used for the assembly of pEND-Z11 or pEND-ZY	Contains sgRNA-2	This study*
pNB-Rep	238996	Spectinomycin	pUC	Node B carrying the sfGPF reporter that can be used for the assembly of pEND-11Y, pEND-Y11,	Contains sfGFP -MarAn10 downstream of a strong constitutive promoter and a binding site for sgRNA-1	This study*

Name	Addgene ID	Resistance	Ori	Intended use	Comments	Source
				pEND-Z11 or pEND-ZY		
pNC-011	238997	Spectinomycin	pUC	Empty Node C that can be used for the assembly of pEND-Y11 and pEND-Z11	Empty	This study*
pNC-Y	238998	Spectinomycin	pUC	Node C carrying sgRNA-1 that can be used for the assembly of pEND-11Y or pEND-ZY	sgRNA-1 downstream of a weak constitutive promoter and a binding site for sgRNA-2	This study*
pEND-11Y	238999	Kanamycin	ColA	Final circuit with -Ara: OFF, +Ara: OFF behaviour	Circuit with no sfGFP expression (-Ara: OFF, +Ara: OFF)	This study*
pEND-Y11	239000	Kanamycin	ColA	Final circuit with -Ara: ON, +Ara: OFF behaviour	Circuit with sfGFP expression only in the absence of Ara (-Ara: ON, +Ara: OFF)	This study*
pEND-Z11	239001	Kanamycin	ColA	Final circuit with -Ara: ON, +Ara: ON behaviour	Circuit with sfGFP expression (-Ara: ON, +Ara: ON)	This study*
pEND-ZY	239002	Kanamycin	ColA	Final circuit with -Ara: OFF, +Ara: ON behaviour	Circuit with sfGFP expression only in the presence of Ara (-Ara: OFF, +Ara: ON)	This study*

*Derived from a plasmid from the Schaerli lab, Department of Fundamental Microbiology, University of Lausanne, Lausanne, Switzerland.

Supporting Figures

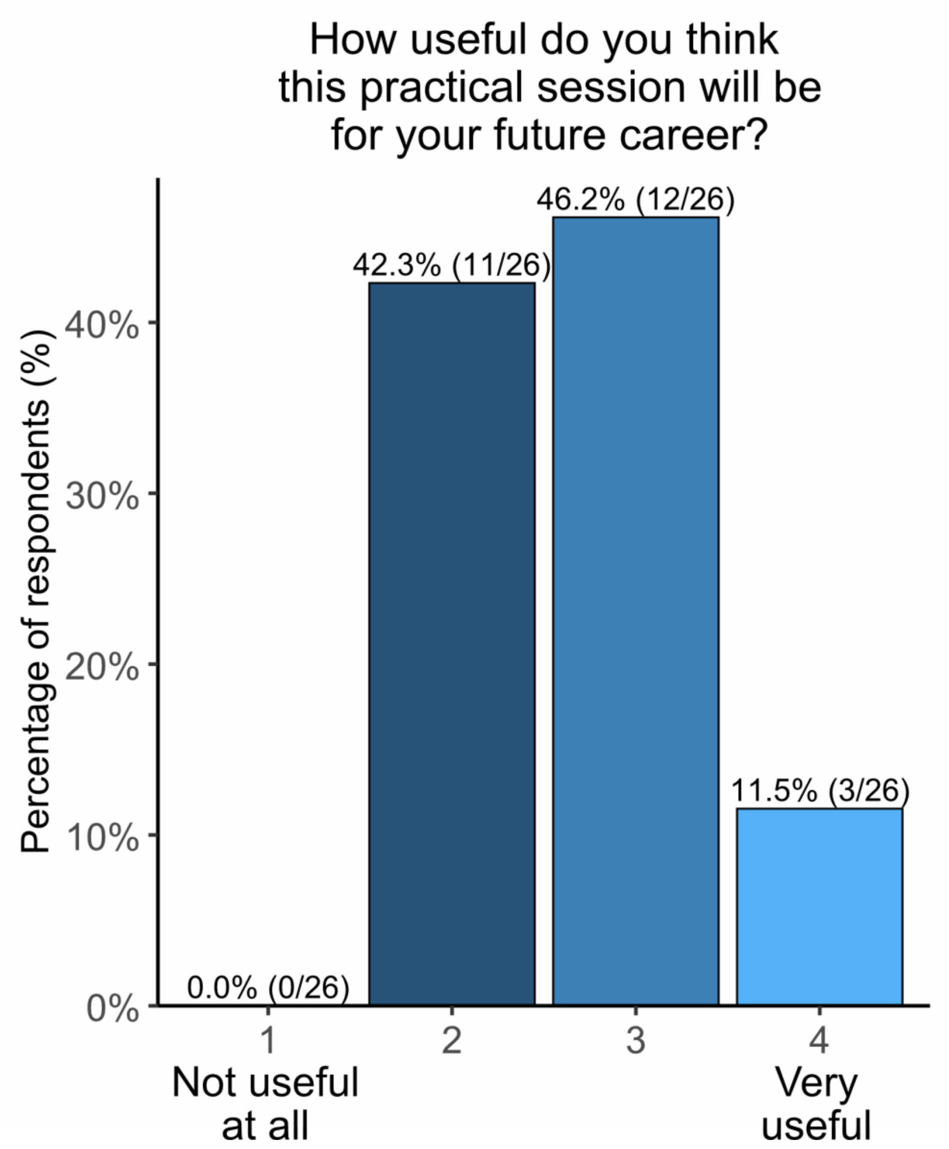

Supporting Figure S1.- Laboratory sessions are mostly perceived as useful for a future career. Survey responses from class participants on how useful they think the practical laboratory session was for their career in a scale from 1 to 4, where 1 stands for "Not useful at all" and 4 stands for "Very useful".

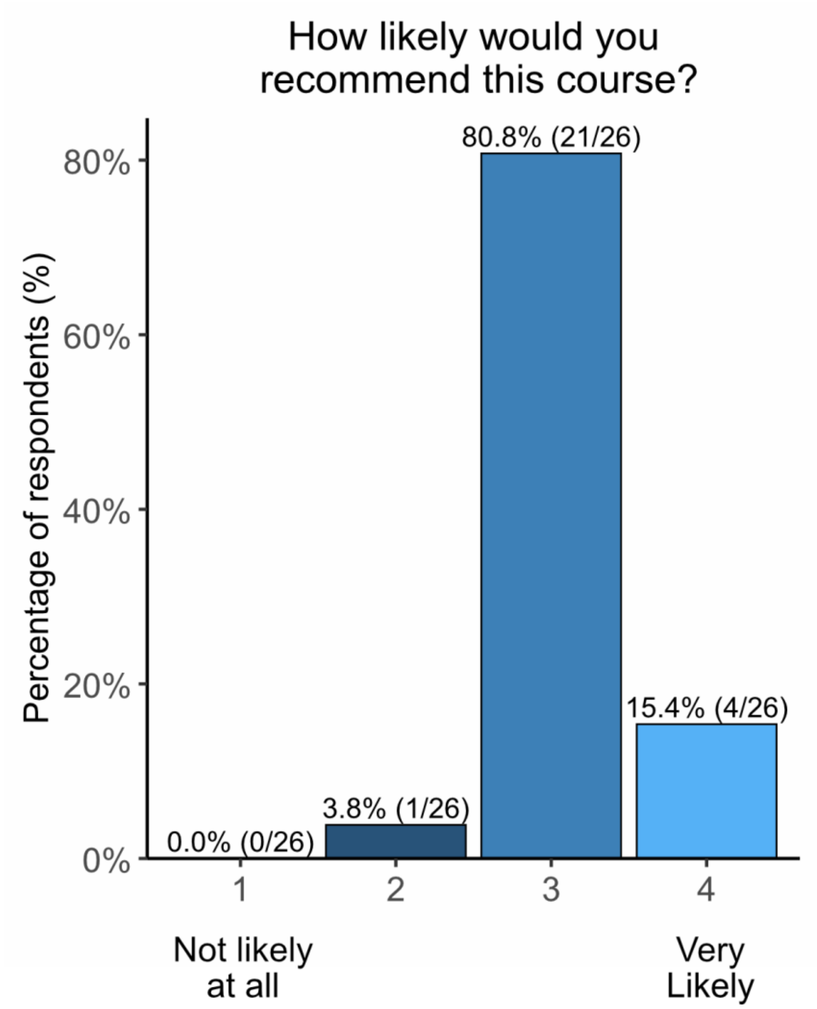

Supporting Figure S2.- The course is recommended for future students. Survey responses from class participants on how likely they would recommend the course in a scale from 1 to 4, where 1 stands for "Not likely at all" and 4 stands for "Very likely".

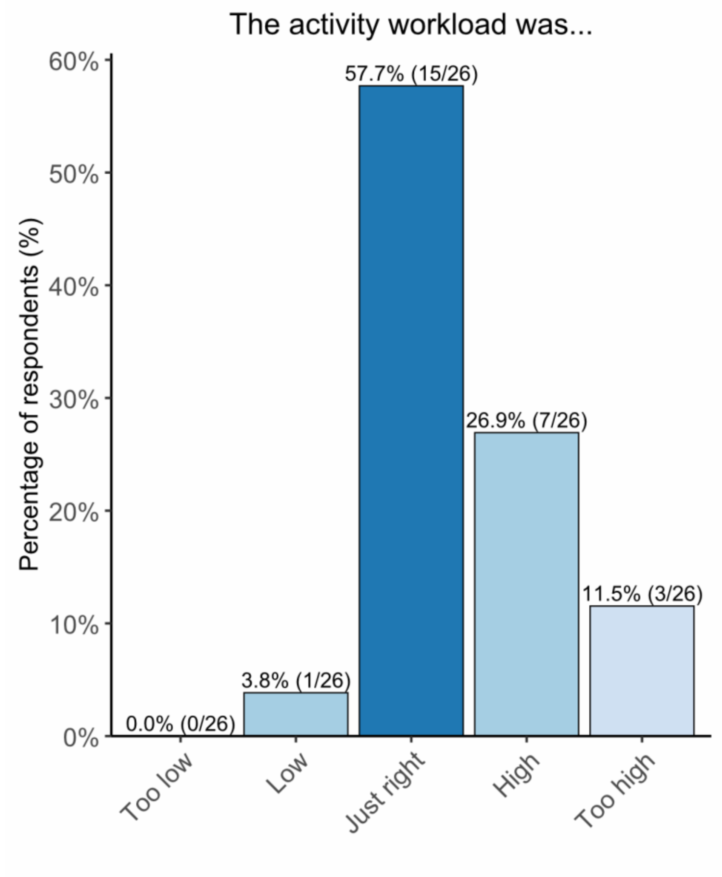

Supporting Figure S3.- Reported activity workload of the preprint-based activity. Survey responses from class participants on whether they considered the activity workload adequate.
