# Supplementary material for: Critical Analysis of Preprints and Inquiry-Based Lessons Improve the Synthetic Biology Learning Experience
Source: ACS Synth Biol. 2025 Aug 15;14(8):2878–84. doi: 10.1021/acssynbio.5c00014 (PMC12362600; doi:10.1021/acssynbio.5c00014)
Supplement: Supplementary file 2 [file sb5c00014_si_002.pdf]

Supporting Information
(Supporting Material S1)

Critical analysis of preprints and inquiry-based lessons improve synthetic biology learning experience

Guillermo Nevot[1*], Marc Güell[1,2] and Javier Santos-Moreno[1*]

[1]Department of Medicine and Life Sciences, Universitat Pompeu Fabra, Barcelona, 08003 Spain
[2]ICREA, Institució Catalana de Recerca i Estudis Avançats, Barcelona, 08003 Spain

*Correspondence to Guillermo Nevot (guillermo.nevot@upf.edu) and Javier Santos-Moreno (javier.santos@upf.edu)

This PDF file includes:

Letter to Editor template given to students for the first assignment of the preprint-based learning activity

Title

Name Surname, … and Name Surname*

Department of XXXXXX, University Pompeu Fabra, [Address]

KEYWORDS: first, second, third, fourth, fifth

ABSTRACT: Lorem ipsum dolor sit amet consectetur adipiscing elit sed do eiusmod tempor incididunt ut labore et dolore magna aliqua Ut enim ad minim veniam quis nostrud exercitation ullamco laboris nisi ut aliquip ex ea commodo consequat Duis aute irure dolor in reprehenderit in voluptate velit esse cillum dolore eu fugiat nulla pariatur Excepteur sint occaecat cupidatat non proident sunt in culpa qui officia deserunt mollit anim id est laborum Lorem ipsum dolor sit amet consectetur adipiscing elit sed do eiusmod tempor incididunt ut labore et dolore magna aliqua Ut enim ad minim veniam quis nostrud exercitation ullamco laboris nisi ut aliquip ex ea commodo consequat Duis aute irure dolor in reprehenderit in voluptate velit esse cillum dolore eu fugiat nulla pariatur Excepteur sint occaecat cupidatat non proident sunt in culpa qui officia deserunt mollit anim id est laborum Lorem ipsum dolor sit amet consectetur adipiscing elit sed do eiusmod tempor incididunt ut labore et dolore magna

Lorem ipsum dolor sit amet, consectetur adipiscing elit. Nullam magna metus, convallis sed semper sed, maximus nec lorem. Praesent sit amet tortor condimentum, iaculis sapien non, viverra sem. Cras sit amet placerat tellus, sed iaculis ex. In ac sagittis mauris. Sed nec nisi lacus. Integer semper ante libero, sed porttitor ligula sagittis vel. Class aptent taciti sociosqu ad litora torquent per conubia nostra, per inceptos himenaeos. Curabitur ut enim lobortis, maximus metus eget, egestas libero. Sed eu tellus sit amet purus condimentum sodales.

Nullam lorem nulla, bibendum sit amet ex a, tincidunt elementum massa. In vel sollicitudin nibh, non finibus turpis. Donec id molestie ipsum. Vivamus tristique pulvinar blandit. Etiam accumsan porttitor augue, ac sagittis velit rhoncus sit amet. In egestas sapien elit. In rutrum augue nec posuere tincidunt. Suspendisse potenti. Nunc suscipit tortor bibendum ex blandit, at tincidunt magna tempus. Vestibulum feugiat dictum dignissim. Praesent facilisis urna sollicitudin, porta nisl eu, euismod ipsum.

Sed at lorem neque. Nulla in augue blandit, commodo arcu non, fermentum neque. Praesent posuere augue eget lorem tempor imperdiet. In accumsan nisl in volutpat feugiat. Nam dictum porta turpis, ut aliquam nisi efficitur sed. Vestibulum pretium elit at lectus consequat bibendum. Nullam faucibus ante nec dui ullamcorper, id facilisis lorem imperdiet. Phasellus venenatis tellus nec nisl posuere, a pharetra sem varius. Nam faucibus purus nunc, ut faucibus urna porttitor eget. Sed imperdiet vitae massa at fermentum. Phasellus ultrices nunc ante, a cursus orci vestibulum molestie. Vestibulum ut felis bibendum, euismod tortor sed, gravida orci. In vel efficitur dui, ac lacinia tortor. Aliquam nulla tortor, efficitur vitae tincidunt eget, dapibus in enim.

Mauris eu est a lacus ullamcorper congue vitae non mauris. Praesent fermentum lobortis sem placerat eleifend. Pellentesque velit sapien, gravida quis vestibulum ut, porttitor ac augue. Ut nunc est, ornare vel sollicitudin at, scelerisque vitae lacus. Suspendisse non porttitor dolor. Pellentesque nulla risus, bibendum a arcu non, tristique gravida velit. Aenean a hendrerit arcu. Etiam cursus hendrerit sapien, ac fermentum urna vestibulum in. Aliquam in nisl lobortis, laoreet justo vitae, aliquam quam. Nulla at erat id massa venenatis gravida sit amet sit amet sapien. Etiam placerat, tortor vel cursus suscipit, felis dolor pharetra eros, eget venenatis lacus tortor eget tellus. Nullam tincidunt tempor enim ac elementum. Cras sollicitudin ullamcorper justo ac efficitur. Morbi vel arcu enim. Donec sit amet risus ut erat posuere cursus ut sit amet nulla.

Donec interdum euismod nunc eu bibendum. Nunc ut sapien aliquet, malesuada dolor a, scelerisque urna. Nunc feugiat sapien ornare felis auctor, quis molestie ipsum tincidunt. Cras elementum ornare dui ut finibus. Mauris euismod dui lobortis fringilla volutpat. Etiam at sem at ex iaculis tincidunt. Nulla feugiat pretium ante. Vivamus quis congue dolor. Pellentesqu Lorem ipsum dolor sit amet, consectetur adipiscing elit. Nullam magna metus, convallis sed semper sed, maximus nec lorem. Praesent sit amet tortor condimentum, iaculis sapien non, viverra sem. Cras sit amet placerat tellus, sed iaculis ex. In ac sagittis mauris. Sed nec nisi lacus. Integer semper ante libero, sed porttitor ligula sagittis vel. Class aptent taciti sociosqu ad litora torquent per conubia nostra, per inceptos himenaeos. Curabitur ut enim lobortis, maximus metus eget, egestas libero. Sed eu tellus sit amet purus condimentum sodales.

Nullam lorem nulla, bibendum sit amet ex a, tincidunt elementum massa. In vel sollicitudin nibh, non finibus turpis. Donec id molestie ipsum. Vivamus tristique pulvinar blandit. Etiam accumsan porttitor augue, ac sagittis velit rhoncus sit amet. In egestas sapien elit. In rutrum augue nec posuere tincidunt. Suspendisse potenti. Nunc suscipit tortor bibendum ex blandit, at tincidunt magna tempus.

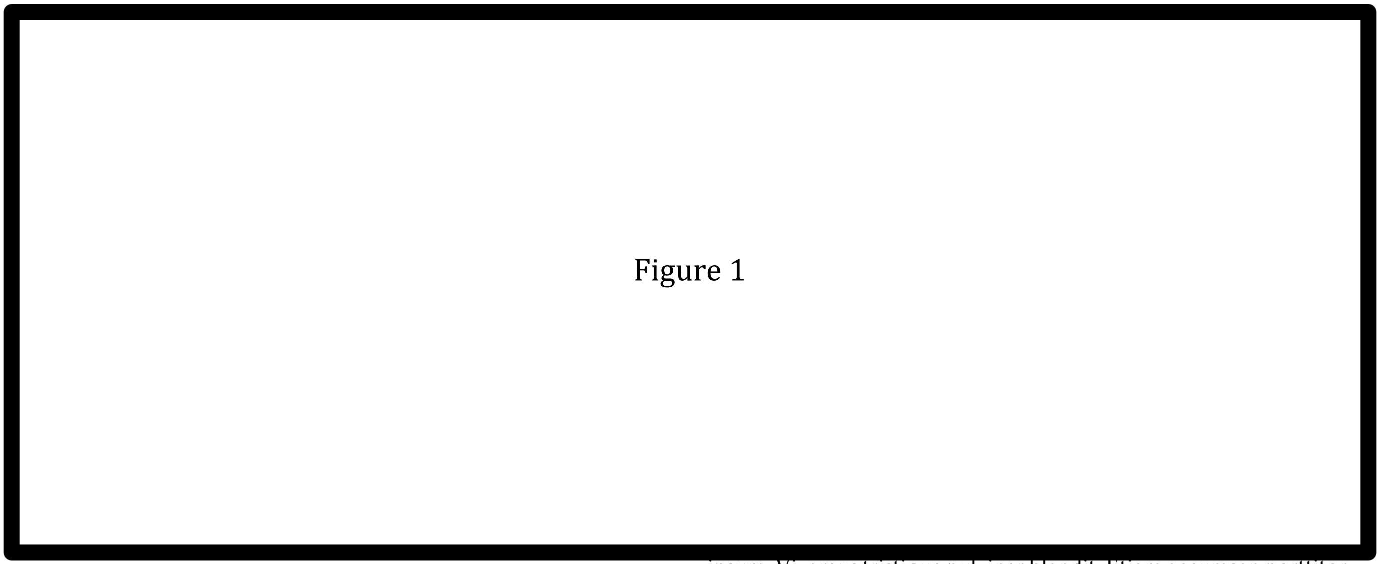

Vestibulum feugiat dictum dignissim. Praesent facilisis urna sollicitudin, porta nisl eu, euismod ipsum.

Sed at lorem neque. Nulla in augue blandit, commodo arcu non, fermentum neque. Praesent posuere augue eget lorem tempor imperdiet. In accumsan nisl in volutpat feugiat. Nam dictum porta turpis, ut aliquam nisi efficitur sed. Vestibulum pretium elit at lectus consequat bibendum. Nullam faucibus ante nec dui ullamcorper, id facilisis lorem imperdiet. Phasellus venenatis tellus nec nisl posuere, a pharetra sem varius. Nam faucibus purus nunc, ut faucibus urna porttitor eget. Sed imperdiet vitae massa at fermentum. Phasellus ultrices nunc ante, a cursus orci vestibulum molestie. Vestibulum ut felis bibendum, euismod tortor sed, gravida orci. In vel efficitur dui, ac lacinia tortor. Aliquam nulla tortor, efficitur vitae tincidunt eget, dapibus in enim.

Mauris eu est a lacus ullamcorper congue vitae non mauris. Praesent fermentum lobortis sem placerat eleifend.

RESULTS AND DISCUSSION

Lorem ipsum dolor sit amet, consectetur adipiscing elit. Nullam magna metus, convallis sed semper sed, maximus nec lorem. Praesent sit amet tortor condimentum, iaculis sapien non, viverra sem. Cras sit amet placerat tellus, sed iaculis ex. In ac sagittis mauris. Sed nec nisi lacus. Integer semper ante libero, sed porttitor ligula sagittis vel. Class aptent taciti sociosqu ad litora torquent per conubia nostra, per inceptos himenaeos. Curabitur ut enim lobortis, maximus metus eget, egestas libero. Sed eu tellus sit amet purus condimentum sodales.

Nullam lorem nulla, bibendum sit amet ex a, tincidunt elementum massa. In vel sollicitudin nibh, non finibus turpis. Donec id molestie ipsum. Vivamus tristique pulvinar blandit. Etiam accumsan porttitor augue, ac sagittis velit rhoncus sit amet. In egestas sapien elit. In rutrum augue nec posuere tincidunt. Suspendisse potenti. Nunc suscipit tortor bibendum ex blandit, at tincidunt magna tempus. Vestibulum feugiat dictum dignissim. Praesent facilisis urna sollicitudin, porta nisl eu, euismod ipsum.

Sed at lorem neque. Nulla in augue blandit, commodo arcu non, fermentum neque. Praesent posuere augue eget lorem tempor imperdiet. In accumsan nisl in volutpat feugiat. Nam dictum porta turpis, ut aliquam nisi efficitur sed. Vestibulum pretium elit at lectus consequat bibendum. Nullam faucibus ante nec dui ullamcorper, id facilisis lorem imperdiet. Phasellus venenatis tellus nec nisl posuere, a pharetra sem varius. Nam faucibus purus nunc, ut faucibus urna porttitor eget. Sed imperdiet vitae massa at fermentum. Phasellus ultrices nunc ante, a cursus orci vestibulum molestie. Vestibulum ut felis bibendum, euismod tortor sed, gravida orci. In vel efficitur dui, ac lacinia tortor. Aliquam nulla tortor, efficitur vitae tincidunt eget, dapibus in enim.

Mauris eu est a lacus ullamcorper congue vitae non mauris. Praesent fermentum lobortis sem placerat eleifend. Pellentesque velit sapien, gravida quis vestibulum ut, porttitor ac augue. Ut nunc est, ornare vel sollicitudin at, scelerisque vitae lacus. Suspendisse non porttitor dolor. Pellentesque nulla risus, bibendum a arcu non, tristique gravida velit. Aenean a hendrerit arcu. Etiam cursus hendrerit sapien, ac fermentum urna vestibulum in. Aliquam in nisl lobortis, laoreet justo vitae, aliquam quam. Nulla at erat id massa venenatis gravida sit amet sit amet sapien. Etiam placerat, tortor vel cursus suscipit, felis dolor pharetra eros, eget venenatis lacus tortor eget tellus. Nullam tincidunt tempor enim ac elementum.

REFERENCES

1. Lorem, I.; Ipsum, D.; Dolor, S.; Dolor sit amet consectetur adipiscing elit sed do eiusmod tempor incididunt ut labore et dolore magna aliqua Ut enim ad minim veniam quis nostrud exercitation ullamco laboris nisi ut aliquip ex ea commodo consequat. Journalum Lorem 2025, 1 (1), 1-2.

2. Lorem, I.; Ipsum, D.; Dolor, S.; Sit amet consectetur adipiscing elit sed do eiusmod tempor incididunt ut labore et dolore magna aliqua Ut enim ad minim veniam quis nostrud exercitation ullamco laboris nisi ut aliquip ex ea commodo consequat dolor. Lorem Journal 2025, 1 (1), 1-2.
3. Lorem, I.; Ipsum, D.; Dolor, S.; Amet consectetur adipiscing elit sed do eiusmod tempor incididunt ut labore et dolore magna aliqua Ut enim ad minim veniam quis nostrud exercitation ullamco laboris nisi ut aliquip ex ea commodo consequat dolor sit. Ipsum Biol 2025, 1 (1), 1-2.
4. Lorem, I.; Ipsum, D.; Dolor, S.; Consectetur adipiscing elit sed do eiusmod tempor incididunt ut labore et dolore magna aliqua Ut enim ad minim veniam quis nostrud exercitation ullamco laboris nisi ut aliquip ex ea commodo consequat dolor sit amet. Dolor Res 2025, 1 (1), 1-2.
5. Lorem, I.; Ipsum, D.; Dolor, S.; Adipiscing elit sed do eiusmod tempor incididunt ut labore et dolore magna aliqua Ut enim ad minim veniam quis nostrud exercitation ullamco laboris nisi ut aliquip ex ea commodo consequat dolor sit amet consectetur. Amet Commun 2025, 1 (1), 1-2.
6. Lorem, I.; Ipsum, D.; Dolor, S.; Elit sed do eiusmod tempor incididunt ut labore et dolore magna aliqua Ut enim ad minim veniam quis nostrud exercitation ullamco laboris nisi ut aliquip ex ea commodo consequat dolor sit amet consectetur adipiscing. Journalum Lorem 2025, 1 (1), 1-2.
7. Lorem, I.; Ipsum, D.; Dolor, S.; Sed do eiusmod tempor incididunt ut labore et dolore magna aliqua Ut enim ad minim veniam quis nostrud exercitation ullamco laboris nisi ut aliquip ex ea commodo consequat dolor sit amet consectetur adipiscing elit. Lorem Journal 2025, 1 (1), 1-2.
8. Lorem, I.; Ipsum, D.; Dolor, S.; Do eiusmod tempor incididunt ut labore et dolore magna aliqua Ut enim ad minim veniam quis nostrud exercitation ullamco laboris nisi ut aliquip ex ea commodo consequat dolor sit amet consectetur adipiscing elit sed. Ipsum Biol 2025, 1 (1), 1-2.
9. Lorem, I.; Ipsum, D.; Dolor, S.; Eiusmod tempor incididunt ut labore et dolore magna aliqua Ut enim ad minim veniam quis nostrud exercitation ullamco laboris nisi ut aliquip ex ea commodo consequat dolor sit amet consectetur adipiscing elit sed do. Dolor Res 2025, 1 (1), 1-2.
10. Lorem, I.; Ipsum, D.; Dolor, S.; Tempor incididunt ut labore et dolore magna aliqua Ut enim ad minim veniam quis nostrud exercitation ullamco laboris nisi ut aliquip ex ea commodo consequat dolor sit amet consectetur adipiscing elit sed do eiusmod. Amet Commun 2025, 1 (1), 1-2.
11. Lorem, I.; Ipsum, D.; Dolor, S.; Incididunt ut labore et dolore magna aliqua Ut enim ad minim veniam quis nostrud exercitation ullamco laboris nisi ut aliquip ex ea commodo consequat dolor sit amet consectetur adipiscing elit sed do eiusmod tempor. Journalum Lorem 2025, 1 (1), 1-2.
12. Lorem, I.; Ipsum, D.; Dolor, S.; Ut labore et dolore magna aliqua Ut enim ad minim veniam quis nostrud exercitation ullamco laboris nisi ut aliquip ex ea commodo consequat dolor sit amet consectetur adipiscing elit sed do eiusmod tempor incididunt. Lorem Journal 2025, 1 (1), 1-2.
