# Supplementary material for: Critical Analysis of Preprints and Inquiry-Based Lessons Improve the Synthetic Biology Learning Experience
Source: ACS Synth Biol. 2025 Aug 15;14(8):2878–84. doi: 10.1021/acssynbio.5c00014 (PMC12362600; doi:10.1021/acssynbio.5c00014)
Supplement: Supplementary file 4 [file sb5c00014_si_004.pdf]

Supporting Information
(Supporting Material S3)

Critical analysis of preprints and inquiry-based lessons improve synthetic biology learning experience

Guillermo Nevot[1*], Marc Güell[1,2] and Javier Santos-Moreno[1*]

[1]*Department of Medicine and Life Sciences, Universitat Pompeu Fabra, Barcelona, 08003 Spain*
[2]*ICREA, Institució Catalana de Recerca i Estudis Avançats, Barcelona, 08003 Spain*

*Correspondence to Guillermo Nevot (guillermo.nevot@upf.edu) and Javier Santos-Moreno (javier.santos@upf.edu)

This PDF file includes:

Teaching protocol for instructors wishing to reproduce the activity in their respective centres where all the laboratory sessions steps are explained in detail

Universitat Pompeu Fabra Barcelona
Facultat de Ciències de la Salut i de la Vida

Advanced Synthetic Biology

Practical session

Universitat Pompeu Fabra Barcelona
Facultat de Ciències de la Salut i de la Vida

Introduction for instructors

In the following teaching session, students are going to build a series of CRISPR interference (CRISPRi) circuits with different GFP fluorescence outputs in the presence of an inducer (arabinose). However, the exact identity of the genetic parts used will initially be unknown for the students, and they will have to deduce which CRISPRi circuit they have built based on final circuit behaviour both in the absence and presence of arabinose. The plasmids used in this practicum correspond to the ones illustrated below:

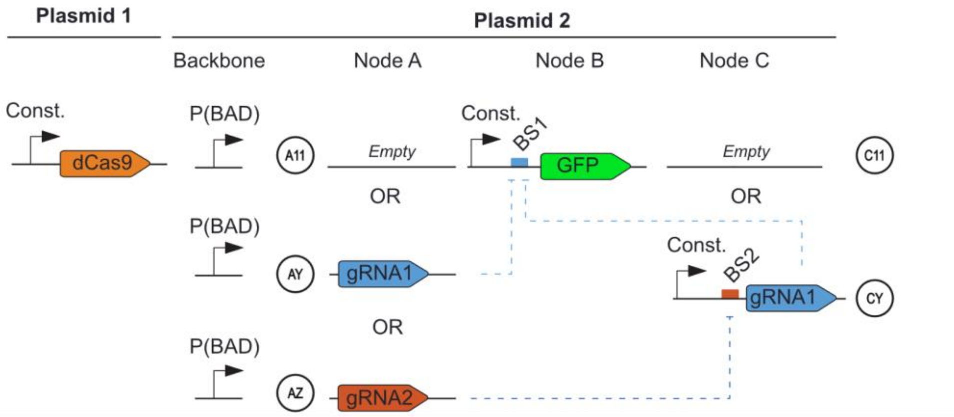

Logic Table

	Arabinose –	Arabinose +
A11-C11	1	1
AY-C11	1	0
AZ-C11	1	1
A11-CY	0	0
AY-CY	0	1
AZ-CY	0	1

Plasmid 1 is already constructed, and students will have to build several versions of plasmid 2 by combining four different fragments derived from four plasmid types: a backbone coming from pBackB plasmid, a node A coming from a “pNA”-type plasmid (either pNA-011, pNA-Y, or pNA-Z), a node B plasmid (pNB-Rep), and a node C coming from a “pNC”-type plasmid (pNC-011 or pNC-Y).To do so, students have to select one of the combinations of plasmids in the table above (except for 2 forbidden combinations, to avoid output repetition), perform digestions that generate the appropriate Gibson overlaps, clean up those digestions, perform a Gibson assembly, and transform it into home-made *E. coli* competent cells harbouring a plasmid with constitutively-expressed dCas9 (pJ1996_v2).

When introducing the activity to the students, instructors should be careful to avoid providing information that would readily allow the students to know which is the identity of the parts that they are using. Typically, we draw the schema above representing the plasmids but we avoid writing the names of the plasmids. In parallel, we let students pick a tube containing the backbone plasmid (Addgene plasmid pC-0, that we rename as pBackB to avoid confusion with node C plasmids) and the plasmid for node B (pNB-Rep); for nodes A and C, students have to choose one plasmid among the different options: 3 choices for node A (pNA-011, pNA-Y, pNA-Z), and 2 choices for node C (pNC-011, pNC-Y) - but we ensure that no student chooses one of the forbidden combinations. We avoid mentioning which of the drawn plasmids those names correspond to. As for the truth table, we provide it empty, and it is the students who have to fill it as they figure out the logic of the possible circuits and the corresponding GFP output.

Practical session

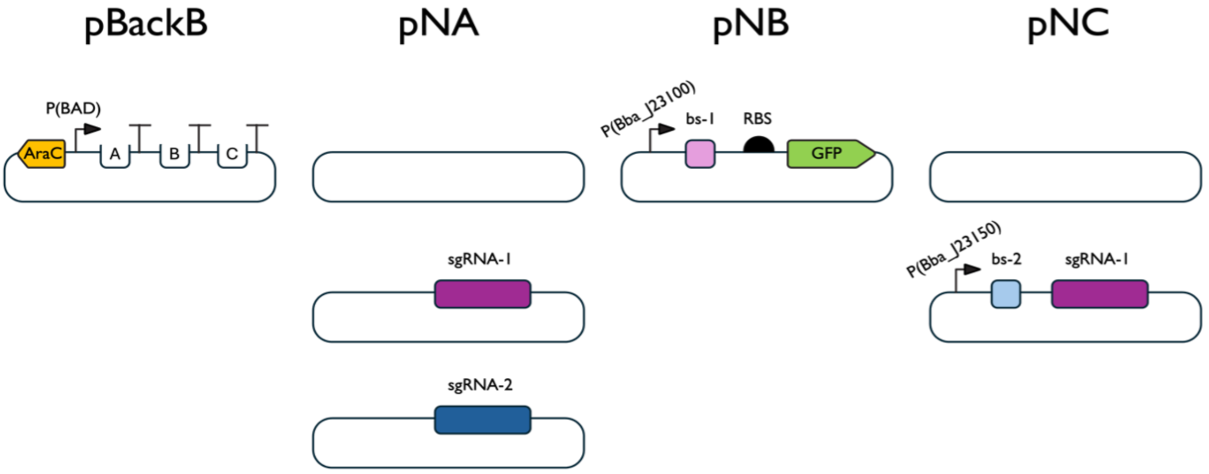

During the following days, you are going to create a series of **CRISPRi circuits** but you are **not going to know which one** until the very end! In the picture above you can see the different plasmids we have that you are going to combine blindly. Try to deduce what will be the output (GFP expression activated or not) for each combination when the circuit is induced or not with arabinose. Try to fill a truth table like this one on your own:

	**Output (GFP)**	
**Combination**	**Ara -**	**Ara +**
Combination 1 (A1-B-C1)	0 or 1 ?	0 or 1 ?
...	...	...

Day 1 — Gibson assembly

Today, we will use Gibson assembly to create a set of CRISPRi circuits. In order to do so, we are going to:

1. Digest the plasmids containing the gRNA and other elements to generate linear DNA suitable for the Gibson assembly.
2. Purify the DNA fragments using a clean up kit.
3. Assemble the CRISPRi circuit using Gibson assembly.

For this teaching session, we have designed the following plasmids that can be combined together using Modular Gibson Assembly (MoGib).

Transcriptional Unit digestion for linearization

In your bench you should have a set of plasmids containing different transcriptional units (TUs). They are labelled with a letter code (NA, NB or NC) indicating the position in the final assembly and an extra code indicating the specific TU. You should also have the final backbone (pBackB) that you also need to linearize for doing the Gibson.

For the next step you need to prepare 4 reactions using the following restriction enzymes:

1. pNA-011, pNA-Z or pNA-Y plasmid — EcoRI and HindIII
2. pNB-GFP plasmid — EcoRI and SalI
3. pNC-011 or pNC-Y plasmid — EcoRI and AscI
4. pBackB plasmid — SacI and KasI

Note: Enzymes used correspond to the following New England Biolabs (NEB) Catalog numbers: EcoRI-HF (#R3101S), HindIII-HF (#R3104S), SalI-HF (#R3104S), AscI (#R0558S), SacI-HF (#R3156S) and KasI (#R0544S).

1. Prepare in one small PCR tube the following reaction:

Reagent	Volume (µl)
Plasmid DNA	1 µg (calculate µl depending on plasmid concentration)
10x CutSmart Buffer	2
Restriction enzyme 1	1
Restriction enzyme 2	1
Water	-
Total	20

2. Incubate in thermocycler 37ºC for 1h.

Digestion clean up

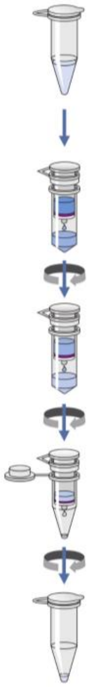

We are going to use the QIAquick PCR purification from Qiagen (#28104) to purify our plasmid digestions. To do so:

1. Add 5 volumes of buffer PB into the digestion. Mix thoroughly.
2. Transfer the whole solution to a Quiaquick spin column.
3. Centrifuge at max. speed for 1 min and discard the follow-through.
4. Add 750 of buffer PE into the column.
5. Centrifuge at max. speed for 1 min and discard the follow-through.
6. Centrifuge again for 2min to dry the column.
7. Transfer the column to a new properly labelled 1.5ml tube.
8. Add 20ul of water to the centre of the column.
9. Wait for 1 minute.
10. Centrifuge at max. speed for 1 min.
11. Measure concentration with Nanodrop.

Gibson assembly

For the Gibson reaction, you need to calculate the moles of vector that you are adding (pBackB) and add 3 times more moles of the rest of the DNA parts. We will use the NEBuilder® HiFi DNA Assembly Master Mix (#E2621L) from NEB. You can use this formula to facilitate your calculations:

$$required\ mass\ insert\ (ng) = desired\ insert/vector\ ratio\ * \\ mass\ of\ vector\ (ng)\ * ratio\ of\ insert/vector\ length$$

It is critical to prepare the full reaction on ice

1. Prepare the following in a 1.5ml tube on ice:

Reagent	Size (bp)	Volume (ul)
linear pBackB	3300	30 ng
linear pNA	520	3x molar ratio
linear pNB-GFP	1360	3x molar ratio
linear pNC	520	3x molar ratio
**Gibson assembly mix**	**-**	**pBackB + pNA + pNB-GFP + pNC**

2. In a thermoblock, incubate the tube at 50ºC for 1h.

Bacterial culture preparation

In order to have growing cultures for tomorrow's experiment, we have to prepare an inoculum today. The strain that we are going to grow (MK01, Kogenaru & Tans, *J Biol Eng* 8, 2, 2014) harbours constitutively-expressed dCas9 (by having the plasmid pJ1996_v2 that confers Spectinomycin resistance). We refer to this strain as M003.

1. Take a bacterial culture glass tube.
2. Pipet 3 mL of LB medium.
3. Add the appropriate amount of antibiotic (Spectinomycin) to dilute 1000-fold (Final concentration of Spectinomycin is 50 µg/mL).
4. Touch the glycerol bacterial stock or plate with a sterile tip and inoculate the tube you just prepared.
5. Let it grow at 37ºC overnight in the shaking incubator.

Day 2 — Transformation

Transformation in *E. coli*

Remember that the overnight culture of *E. coli* M003 (MK01 strain with the dCas9-expressing plasmid pJ1996_v2) that you inoculated yesterday has been re-inoculated this morning by the instructors (40uL of the overnight culture into 1.4mL of fresh LB media in a 2mL or 10 mL tube during 3-4 hours at 37°C) to provide you with an exponential culture of *E. coli* ready to start with the competent cell preparation.

For the following steps: keep the cells on ice.

1.Transfer the culture to a 15mL tube. Centrifuge 10 min at 4000xg and remove the supernatant without touching the pellet.
2. Add 1mL of ice-cold water, resuspend with the pipette and add ice-cold water up to 10mL. Keep the cells always on ice.
3. Centrifuge 10 min at 4000xg and remove the supernatant without touching the pellet.
4. Add 1mL of ice-cold water, resuspend with the pipette and add ice-cold water up to 10mL. Keep the cells always on ice.
5. Repeat steps 3 and 4 twice more.
6. Remove most of the supernatant and leave ~50uL, then resuspend the pellet by tapping.
7. Add to these cells between **0.5 and 1 uL of plasmid.**
8. Place the mix in an 1 mm electroporation cuvette, make sure that the surface is completely dry, and put it inside the electroporator.
9. Electroporate using the following settings: 1.8 kV 25 µF 200 Ω, (time constant in milliseconds should be between 4 and 6), and rapidly add 300 uL of SOC or LB medium at 37º C.
10. Transfer the mix to a new 1.5 tube and incubate with shaking at 37°C for 1 hour.
11. Plate 150uL of the construct in LB plates with spectinomycin and kanamycin (final concentration of 50 µg/mL for both antibiotics).
12. Plate 150 µL of the constrict in LB plates containing both antibiotics and 0.2% arabinose.

Day 3 — PCR verification and Arabinose induction

To confirm that the transformed bacteria have incorporated the desired DNA construct, we are going to amplify the target DNA with a colony PCR, which is a PCR in which the genetic material used as template in the reaction comes from a bacterial colony that is directly added to the PCR mix, rather than from purified DNA.

PCR verification of gRNA insertion

PCR reaction:

1. Prepare in a PCR tube the following reaction. You have to prepare as many reactions as colonies as you want to test.

Reagent	Volume (µl)
Primer FW (5'-acggcgtcacactttgc-3')	1
Primer RV (5'-gggccgttgcttcacaacg-3')	1
Taq Vazyme (x2)	12.5
H_2O	-
Total	25

2. Touch lightly a colony from the plate with a sterile tip. Introduce the tip in the reaction mix.
3. In a thermocycler, perform the following steps

Temperature	Time	Cycles
95 ºC	10 min	
95 ºC	15 s	|
60 ºC	15 s	| 25x
72 ºC	60 s/kb	|
72 ºC	5 min	
4 ºC	∞	

After obtaining the PCR products, they will be loaded in an agarose gel (1% agarose in 1X TAE buffer supplemented with 10 ul of SYBR™ Safe DNA Gel Stain) to check if the amplicon size corresponds to the expected size.

Gel electrophoresis

1. Place the agarose gel inside the electrophoresis tank and cover it with a 1X TAE buffer.
2. The PCR reaction mix already contains the loading buffer.
3. Load 20ul in one well of the agarose gel.
4. Remember that at least one well per gel is necessary to incorporate a ladder as a guide of the size of our fragments. Load 5ul of the DNA ladder.
5. When everybody has loaded the samples, put the lid to the tank and connect it to the power source at 135V for 30 min.
6. After running the gel, the bands will be visualised under UV light.

GFP Measurement

To evaluate the output of the CRISPRi circuit you have built, place your transformants (both those in the Ara- and in the Ara+ plates) in the UV-transilluminator to observe if the colonies are fluorescent or not. Compare the results with the positive controls provided by the instructors: M003 transformed with pEND-11Y, pEND-Y11, pEND-Z11, or pEND-ZY.
