# Supplementary material for: Critical Analysis of Preprints and Inquiry-Based Lessons Improve the Synthetic Biology Learning Experience
Source: ACS Synth Biol. 2025 Aug 15;14(8):2878–84. doi: 10.1021/acssynbio.5c00014 (PMC12362600; doi:10.1021/acssynbio.5c00014)
Supplement: Supplementary file 3 [file sb5c00014_si_003.pdf]

Supporting Information
(Supporting Material S2)

Critical analysis of preprints and inquiry-based lessons improve synthetic biology learning experience

Guillermo Nevot[1*], Marc Güell[1,2] and Javier Santos-Moreno[1*]

[1]Department of Medicine and Life Sciences, Universitat Pompeu Fabra, Barcelona, 08003 Spain
[2]ICREA, Institució Catalana de Recerca i Estudis Avançats, Barcelona, 08003 Spain

*Correspondence to Guillermo Nevot (guillermo.nevot@upf.edu) and Javier Santos-Moreno (javier.santos@upf.edu)

This PDF file includes:

Editorial Assessment template given to students for the second assignment of the preprint-based learning activity

Article title

Editor summary:

A short summary of the critique explained in your own words as an editor. This helps authors to see if you really understood their point or not.

Relevant references

This is a list of around 3 references that are key to understanding the letter to the editor. Usually, we include the reference and why the editor thinks is relevant for the assessment.

Reviewer reports

Include at least 2 reviewer reports with their complaints about the letter to the editor. You can support or criticize the arguments exposed in the letter but they need to always be justified.

Reviewer #1:

Reviewer major comments

Usually left for major changes in arguments and concerns.

Reviewer minor comments

Small comments regarding how the sentences are written or small complementary details.

Reviewer #2:

Reviewer major comments

Usually left for major changes in arguments and concerns.

Reviewer minor comments

Small comments regarding how the sentences are written or small complementary details.

Reviewer #3:

Reviewer major comments

Usually left for major changes in arguments and concerns.

Reviewer minor comments

Small comments regarding how the sentences are written or small complementary details.

Summary of opinions an editorial decision

Here the editor summarizes the opinion of the reviewers and justifies why, based on the opinion of the reviewers and his own judgment, decides whether to publish or not. The arguments here can go beyond science and comment on audiences, if they are going to be relevant for the journal line etc.

Please choose and justify if you decide to:

- Reject the paper
- Accept provided that some specific revisions are fulfilled.
